# Governance models for historical hospitals: evidence from Italy

**DOI:** 10.1186/s12913-024-10640-w

**Published:** 2024-03-06

**Authors:** Martina Giusti, Ilaria Elisa Vannini, Niccolò Persiani

**Affiliations:** Department of Experimental and Clinical Medicine, University of Florence, Florence, Italy

**Keywords:** Governance, Cultural heritage, Historical hospital, Network

## Abstract

Many hospitals and health care organizations over the centuries have inherited handcrafts of artistic value, objects of worships, donations from pilgrims, votive offerings, legates as a result of their centuries-old activity in the social and health care of their community. The value of these artistic assets and the role in the territory of these hospitals have transformed them into cultural heritage over time, introducing the issue of the coexistence of cultural and care management. Therefore, the aim of this paper is to identify governance models of what we will call *historical hospitals*, that is, hospitals that because of their history, their assets, and their artistic heritage, far from being places only dedicated to provision of health care services represent cultural heritage. To pursue this research objective, five Italian *historical hospitals* were selected and identified as significant *case studies* for their historic and artistic relevance. Through the analysis of these case studies the paper identifies models of governance of *historical hospitals* and the reasons of their adoption.

**Supplementary Information:**

The online version contains supplementary material available at 10.1186/s12913-024-10640-w.

## Introduction

Many hospitals and health care organizations, in the centuries, have inherited handcrafts of artistic value, objects of worships, donations from pilgrims, votive offerings, legates as result of their centuries-old activity taking social and health care of their community. Overtime, collections of medical instruments, health volumes, furnishings, essays, clinical documents have also been formed. At the same time, monumental buildings, churches, and ancient pharmacies enriched this heritage. Finally, in some cases “old” goods and buildings, which have been recognized over time for their architectural and artistic features as evidence of past times, have simply arrived to date.

Unfortunately, often, part of this heritage accumulated over the centuries has been lost, dispersed or disrupted. This heritage was not only removed from its original function but also from the enjoyment of this by community [1, 2]. Sometime, instead, the artistic assets accumulated by the hospitals and health care organizations in their histories were dislocated in separate cultural institutions or museums, because their management and enhancement appeared irreconcilable with health purposes [3]. Other times hospitals and health care organizations have promoted museum itineraries into their building, opened both to the internal and external public in order to preserve the original health destination [4–7]. In this latter case, the coexistence between cultural and health assets has subjected health institutions to administrative, organizational, and financial difficulties because each kind of heritage has its regulations, and its specific valorisation needs.

Finally, there are the ones that we can define *“historical hospitals”.* For their history, their assets and their artistic heritage, far from being places only dedicated to provision of health care services, these hospitals represented real cultural heritage, intending for cultural heritage something that population has started to identify as a reflection and expression of its values, beliefs, knowledge and traditions (Framework Convention of Council of Europe on the value of cultural heritage for society) [8–10].

In these institutions, in relation to the additional scope and needs of cultural heritage’s conservation and enhancement the coexistence of historical and artistic heritage within a high-tech asset involves three orders of reflections.

First of all, the role and the contribution of the presence of historical-artistic-cultural assets in the achievement of the distinctive mission of health institutions must be clarifying. The same World Health Organization defines art as effective tool for the promotion of well-being and correct lifestyles [11], for treatment support in the field of art therapy [12, 13] or for the humanization of care [14]. In second instance, in historical hospitals the existence of a further mission of management, conservation and enhancement of the own cultural heritage in addition to the distinctive one of health care promotion and recovery makes it necessary to reflect deeply on the governance model to be adopted. Finally, the two types of purposes should be reconciled by the definition of corporate governance, management strategy and financing rules for the most efficient use of the available resources.

These challenges involve finding a governance model of the *historical hospital* that integrates both missions, putting together the potential of cultural resources with the provision of safe, innovative, and humanized health care services.

The aim of this work is to identify the governance models in *historical hospitals* and investigate them based on their ability to efficiently combine the goals of health care with those of conservation and enhancement of a cultural heritage.

## Framework

In the field of cultural heritage management, the concept of governance [15–17] has often been identified with so-called *cultural governance*. The specific concept of cultural governance was investigated by different perspectives in the literature.

First, it was designed as a tool for cultural policymaking applied by different institutions or organizations to improve citizens’ accessibility to art and creative activities [18–24]. In the 1960s UNESCO called cultural policy “a way to both talk about and do something in a certain manner, according to certain principles” [25–28]. These policies change in each country in relation to the processes, laws, regulations, and institutions chosen by each government to support and promote diverse creative expressions of all types of arts [29–36].

On the other hand, the cultural governance has been analysed in relation to the assumed institutional assets, including relations with relevant institutional stakeholders [37–39]. Since the cultural heritage is considered a public good, the roles and activities of institutions deputed to its conservation and enhancement (i.e., municipalities, autonomous institutions, or supervision organizations) are determined by public legislation.

Business economics scholars, instead, have focused their attention on the corporate governance applied to cultural organizations and institutions [40–45]. This was followed by the search for governance models capable of ensuring the efficient pursuit of conservation and enhancement of managed cultural heritage by engaged organizations and institutions [9, 10]. The identification of these governance models is very complex due to the sectorial competences required for the conservation and enhancement of the cultural heritage and the usual co-responsibility by public and private organizations in the management of cultural heritage.

For these reasons, the identification of governance models for cultural heritage required a specific scheme of analysis of the solutions adopted [46, 47]. Literature offers [48] an interesting and complete overview of cultural heritage governance models, applied in the Italian context but referrable elsewhere. In this case a matrix relates the legal nature of the organizations deputed to decision-making and the organizational levels of reference. It permits an easy classification of the various governance models and the related actions for the achievement of the purposes of cultural heritage’s conservation and enlargement by institutions.

In cultural heritage governance the coexistence of public and private entities, each with different purposes, has proven particularly effective in the study of the conservation and enhancement of the so-called corporate collections [49, 50], i.e., art or monumental complexes owned by companies operating in a wide variety of sectors. In this case, the presence of a cultural heritage leads to the definition of a governance model both for the conservation and enhancement of the owned cultural heritage according to the company’s main purposes [51].

While the literature has studied governance models of corporate collections in various sectors, including banks and local authorities [52–54], the study of these governance models in the health care sector, particularly in what we have called *historical hospitals*, appears less frequent.

The cultural heritage owned by hospitals and health care organizations is, in fact, investigated mainly from the perspective of the history of medicine [55–57] or as a collection of artifacts, which can be used in art therapy programs [12, 13, 58] or for humanisation of care [14, 59]. Only few studies are devoted to the study of management disputes [60], conservation and enhancement problems [61] or corporate governance issues related to the ownership of corporate collections by hospitals and health care organizations. This is particularly relevant in a context, like the Italian one, where historically “cultural policy has focused more on the preservation of heritage sites and less on the access to them” [35, 36]. In the health care sector, the governance issues deserve specific attention and require in-depth reflection in order to identify the most appropriate governance models for managing the cultural heritage owned by healthcare institutions.

This paper aims to fill the knowledge gap on the topic of cultural heritage corporate governance and related governance models in *historical hospitals*. Indeed, in *historical hospitals* the daily commitment to the provision of complex healthcare services to thousands of citizens coexists with the responsibility to conserve and enhance a cultural heritage that is a common asset, not only of the corporate but of the entire community.

## Material and method

To pursue the objectives of this paper, five *historical hospitals* were selected and identified as significant case studies for their relevance in the historical, health, and artistic contexts [62]. All of them are Italian and therefore are strictly related to the Italian cultural and healthcare policy context [63]. In Italy the word valorisation indicates a combination of promotion and exploitation [35] and tensions between public provision and outsourcing are an important aspect of the cultural policy [34] along with the impact of public funding cuts to arts and culture.

In particular, the following case studies have been chosen.

The *Santa Maria Nuova Hospital* in Florence is considered the oldest hospital in the world still in activity at the place of foundation [56, 57]. It was founded in 1288 by Folco Portinari with the aim of providing a healthy and rich growing to medieval Florence. This hospital represented an early and effective example of civic health care, the model of which inspired major European hospitals of the time. At present, in the *Santa Maria Nuova Hospital* high-tech wards and complex day and effective outpatients’ services coexist with museum itineraries with priceless works of art, churches and historical cloisters.

The second case study is the *S. Spirito in Sassia Hospital* in Rome [64]. The origins of this hospital dated back to 727 A.D., when the Saxons established through the Schola Saxonum to give hospitality to their countrymen, who came on pilgrimage to the tomb of the Apostle Peter in Rome. Later transformed into a hospital, it became one of the main hospitals in Rome as hospital of the popes. Today this vast monumental complex hosts two innovative hospitals, the frescoed Sistine wards, the historical hospital government building, two libraries, two museums and multiple works of art spread throughout its corridors and waiting rooms that testify its thousand-year history.

*Ca’ Granda Ospedale Maggiore Policlinico Hospital* in Milan [65, 66] was founded by Francesco Sforza in 1456 from the merge of many charitable institutions of the city. Since its inception, it has been proposed as a model of healthcare and organization offered to all citizens, particularly the less well-off. In the past as in the present, the hospital is recognized as one of the main city’s institutions and it attracts donations to support its activities. This leads to the possession both of avant-garde technological, structural, and organizational equipment for the provision of quality health care together and of extraordinary archives and marvellous picture gallery.

The *SS. Giovanni e Paolo Hospital* in Venice [67] is one of the major monumental complexes in the city with its architectural-artistic compendium spanning almost nine centuries. This hospital is also home to the ancient Scuola di San Marco. Today it is engaged both in care and promotion of the health of the population of Venice and, at the same time, in the dissemination of moral and medical-scientific contribution of this institution to the construction and development of Venetian culture [68].

The last case study is *Santa Maria degli Incurabili Hospital* in Naples [69, 70]. Founded in 1521, it testifies the conduction of a humanitarian and health activity aimed at assisting the incurably ill, the most fragile people in the community, who had no other option of improving their condition than to rely on the generosity of the community starting from the XVI century. Today, this hospital continues its efforts to support the promotion of health in the community by conducting assiduous health education and prevention activities and setting up exhibitions on the history of medicine to transfer to new generations the importance of medicine for the progress and improvement of community conditions [70].

The case analysis was conducted in three phases [71]:

1. *Within case analysis*. Data from each case study were analysed separately to provide a complete picture of the governance model adopted and the management issues addressed. The same data analysis scheme was used for each case. Data were acquired through prior documentary analysis of primary and secondary sources, followed by semi-structured interviews with the cultural heritage management. The research group adopted as primary sources publications, books, and administrative documents (i.e., statutes, deliberations) dealing with the development history of the selected *historical hospitals*. Moreover, this information was integrated with semi-structured interviews to have the current state-of-art on *historical hospitals’* governance models. The semi-structured interviews were focused on the following topics: (i) Historical hospitals and referral entities; (ii) Historical background; (iii) Cultural heritage; (iv) Cultural heritage-Health integration; (v) Governance model; (vi) Organizational model; (vii) Methods of financing. In particular, according to the main scope of the paper, governance section contained questions about: legal configuration; acts, deliberations, statute and other documents related to the *historical hospital*’s configuration; recognized institutional purposes; relationship with local health authorities or other stakeholders.

The interviewees were the general managers of the *historical hospitals* as the first promoters of governance models and the cultural heritage managers as referents and supporters of the initiatives of *historical hospitals*’ valorisation. Interviewees were selected for their professional experience and roles into the historical hospitals and not as patient or member of a sample. Each participant was required to sign a privacy policy document to consent the management of their personal data in compliance with the European (Regulation (EU) n. 679/2016, Regulation (EU) n. 536/2014) and national regulation (Italian Law 2019/2017). The request for the approval of the research by the ethic committee or the institutional review board was required because of the absence of health sensible data related to medical treatment and for research involving human participants [72, 73].

2. *Data reduction*. Descriptions of the governance models of each *historical hospital* were submitted to the interviewees to verify the information used in each case study description, to avoid observer bias;
3. *Cross-case analysis*. Comparisons were made among the five *historical hospitals* to identify the similarities and differences as well as the strengths and weaknesses of each [62].

In order to appreciate current adopted governance models of cultural heritage, the analysis of the documentation provided was supplemented by semi-structured interviews with case studies managers involved in cultural policy-making and cultural governance. This enabled the identification of the problems encountered by the case studies and the policy, management, and governance responses.

The analysis of governance models allowed their collocation in a matrix that, in accordance with the methodology for classifying cultural governance models [48], compares the legal nature of the decision-making entities and the organizational levels of reference.

The adopted governance models of cultural heritage governance were then positioned in a further matrix developed by the research team, which relates the problems encountered in the administration of cultural heritage to the critical issue of valorisation of the cultural heritage itself. This matrix highlights the reasons for adopting each governance model and the type of problem it responds to.

## Results

Based on the information obtained from the analysis of the documentation, provided, the interviews with the managers of the five *hospitals* clearly illustrated the main difficulties that characterize the management of a cultural heritage within a *historical hospital* and that are consequently placed at the basis of the governance choices.

These can be grouped into the following typologies:

- Administrative problems of cultural heritage in healthcare organization to sector-specific rules and regulations, both at national and international level;
- Problems of management, conservation, and enhancement of the same taking into account the main purpose of a health organization, which remains care.
- The first issues are consequence of the specific sector legislation to which the hospitals interviewed are subject and concern the management of specific inventories, personnel management, and the procurement of ad hoc funding (Table 1).

**Table 1:** Critical issues related to the management of cultural heritage in health organisations

Administrative issues	
Inventories management	Inventory regulations applied to public health organizations cover only those assets used for the characteristic provision of the health care services. Offices in charge of managing articulated assets of health organizations, have opted for the coexistence of health inventories and inventories required by the field of cultural heritage management. In fact, personnel employed there often does not have a specific expertise in the field of cultural heritage.
Personnel Management	In Italy, public health organizations are prevented from recruiting professional profiles with specific skills related to the management, conservation, and enhancement cultural heritage, because they are not provided for by the current regulations. This led to the retraining of available personnel in the specific field of cultural heritage.
Procurement/use of ad hoc funding	The health purpose of the funding received from the State constrains the investment of these resources only in health, impeding their use in heritage-related activities.

On the other hand, the difficulties in the conservation and enhancement of cultural heritage are mainly related to the complexity of managing cultural heritage within a hospital and the coexistence of the purposes of its management, conservation, and enhancement with the specific purposes of health organizations of health care and promotion (Table 2).

**Table 2:** Critical issues related to the management of cultural heritage in health organisations

Management, conservation, and enhancement issues	
Coexistence of cultural and health purposes	The integration of cultural heritage promotion activities into health care pathways is difficult due to the lack of specific training on the use of art in health care settings addressed to healthcare professionals. This corresponds to a lack of knowledge about the possible benefits for patients, in terms of health outcomes and humanisation of care, and for caregiver and health professionals, in terms of anxiety and stress reduction. This knowledge gap limits the opportunities for integrating together the purpose of health recovery and promotion and cultural heritage conservation and enhancement. While this was thought out and proposed, logistical constraints related to moving patients and/or moving cultural heritage, the lack of codification of this activity in terms of health services and the absence of dedicated personnel have prevented these initiatives from providing continuity so that they could yield the desired results over time.
Attraction of donations and communication strategies	Fundraising campaigns for the conversation and the enhancement of cultural heritage should be entrusted to professionals with cultural expertise. Moreover, the absence of a specific accounting tool to track these donations flows is another limitation. On the other hand, health promotion and protection communication cannot be borrowed in the cultural sector. The risk is that there is a lack of clarity in the message given.
Limited use of cultural heritage in health care organizations	The movement of drugs and patients within a working hospital is often incompatible with the presence of visitors or tourist flows due to the presence of a cultural heritage exhibition route owned by the health organizations themselves. Relocation of the exhibition, at the same time, disrupts the relationship between the historical site and its memory.

To deal with these problems, four governance models of cultural heritage were identified in the conducted interviews. The first governance model can be called *internal administration*. In the public sector, cultural heritage management has traditionally been entrusted to an office responsible for preservation and management of all corporate assets. In many cases this choice derived from specific regulatory obligations (in Italy, the Cultural Heritage Code, Law 42/2004, art. 30, co. 1 and co. 4), in other cases the mere bureaucratic management of the assets was accompanied by a supervisory activity for their protection. This governance model of cultural heritage does not involve significant additional activities for the institution and focuses its attention on the mere conservation of assets. It represents the initial choice common to all case studies. Even today the *Ca’ Granda Ospedale Maggiore Policlinico Hospital* of Milan and the *SS. Giovanni e Paolo Hospital* in Venice entrust the management of their cultural heritage to an office in charge of managing the entire corporate heritage.

A further and more sophisticated governance model is the establishment of an *autonomous entity* dedicated to the conservation and the enhancement and promotion of cultural heritage. In the *historical hospitals* investigated, this means the creation of a foundation wholly owned by the hospital. In this governance model of cultural heritage, a dedicated corporate strategy is developed even though the governance of the cultural heritage is internal to the owner health organization. Indeed, cultural heritage conservation and enhancement initiatives profoundly influence the image of the health organizations and characterize their identity, tying them to the history of the territory they belong to. The limitations of being subject to the strict rules of the public administration remain as the inability to access healthcare funding. This choice was historically made by the *Santa Maria Nuova Hospital* in Florence, the first foundation of this kind in Italy. In interviews, the historical hospitals of *S. Spirito in Sassia Hospital* in Rome and *SS. Giovanni e Paolo Hospital* in Venice are about to adopt the foundation as a new governance model of their cultural heritages.

A third model, which is an evolution of the previous one, is the *cultural holding* in which valorisation is entrusted to an autonomous entity. This governance model connects *historical hospitals* with the cultural sector of the area in which they are located. The implementation of this model promotes the integration of *historical hospitals* in traditional touristic routes and the creation of new ones aimed at as a further objective of the enhancement of their cultural heritage. This choice has recently been made by the *Santa Maria Nuova Hospital* in Florence, whose foundation assumed the responsibility of also enhancing the cultural heritage of other small *historical hospitals* of Florence region such as the *San Giovanni di Dio Hospital* in Florence, the *Ceppo Hospital* in Pistoia, and the *Misericordia e Dolce Hospital* in Prato, at that time at risk of abandonment.

Finally, we observe the choice of an *integrated partnership*, in which the joint management of several cultural heritages of the public hospital by private and public organizations is associated with the management of private cultural heritage. This is what occurs in *Santa Maria degli Incurabili Hospital* in Naples that, on the strength of the experience gained, has set up an association among both public and private partners. The *integrated partnership* is responsible for the management, conservation, and enhancement of both the cultural heritage owned by public health organizations in the Campania Region and the collection of surgical instruments, health technologies and other things related to the history of medicine in the same Region owned by private individuals. External partners are also involved in developing strategies and policies for the conservation and enhancement of the cultural heritage of several health organizations, often initiating collaborations and synergies among them. Single, central, and superior coordination of the *integrated partnership* offers multiple possibilities. The first is the joint adoption of conservation and enhancement strategies, extending the possible catchment area and encouraging the integration among public *historical hospitals* and private health cultural heritage both into traditional and new tourist routes. The second is the safeguard of the peculiarities of the cultural heritage owned by each health organizations into a joint public-private promotional strategy. The third is the access to funding. However, a critical element could be recognized in the risk of detaching of the network’s activity from the single health organizations, which always retain ownership of their cultural heritage. A possible consequence could be the difficulty of integrating the health purposes with those of cultural heritage conservation and enhancement.

**Fig. 1:**
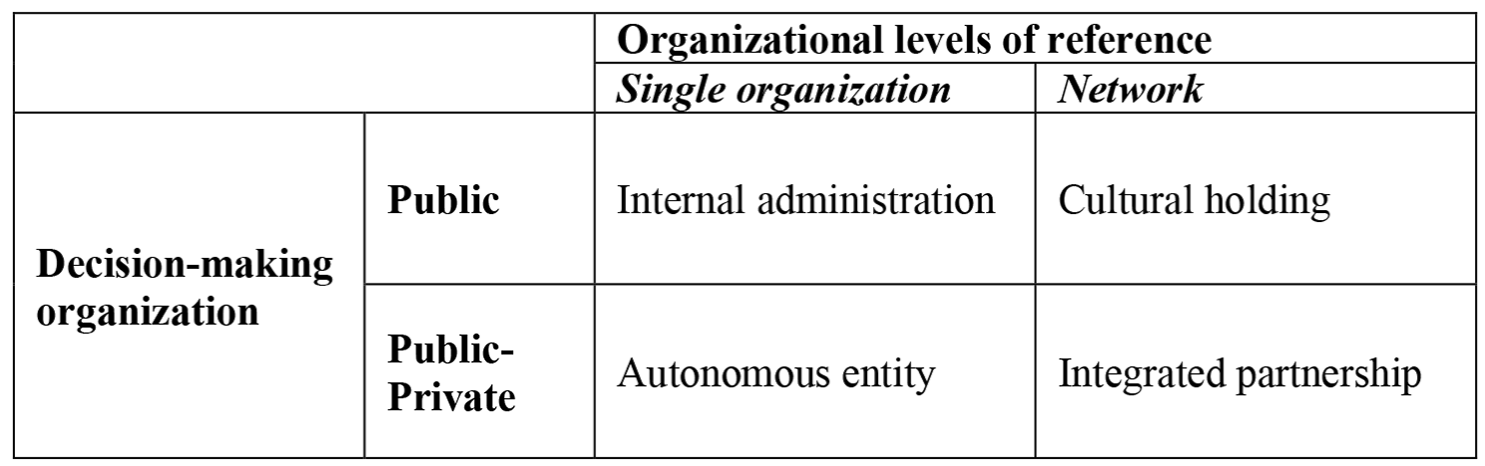
Governance models of historical hospital according to the donato matrix (our elaboration)

The four governance models of cultural heritage adopted by the *historical hospitals* perfectly fit to what the literature has modelled for the governance of cultural heritage. In fact, these governance models can be easily positioned in the following matrix (Fig. 1), which explains the underlying dynamics of these models.

The matrix is obtained by intersecting the legal nature of the decision-making organizations with reference organizational levels. The governance models of public and public-private organizations are intersected with the reference organizational levels at the level of individual organization, micro, or network, macro [48, 74, 75]. Intersecting the two variables, four governance models are identified. They represent the different governance models of cultural heritage by *historical hospitals*: public *internal-administration*; public-private *autonomous entity*; public *cultural holding*; public-private *integrated partnership*.

## Discussion

**Fig. 2:**
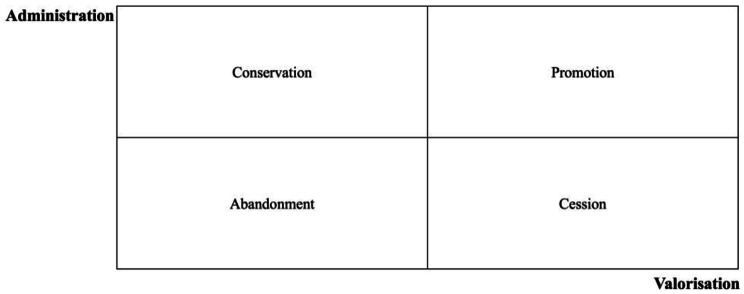
Capability of response of governance models to the problems of administration and valorisation (our elaboration)

The governance models of cultural heritage in *historical hospitals* have been developed to solve some specific issues (Tables 1 and 2). Their ability to respond, on the one hand, to problems of administration and, on the other hand, management, conservation, and enhancement of the cultural heritage by health organizations can be analysed through the matrix represented in Fig. 2. This matrix puts in relation the following variables: the capability of administration and the capability of valorisation, especially in the integration of cultural and health care paths, of the cultural heritage by part of each governance model. From the measurement of these variables (scarce/high) four quadrants, representing four strategies in cultural heritage management, are identified.

We have called these areas:

- *Abandonment* (scarce administration/scarce valorisation). This quadrant collects governance models that combine a low level of administration with a low level of valorisation of the cultural heritage. In this case, the cultural heritage of *historical hospitals* has been abandoned or has been lost.
- *Cession* (high administration/scarce valorisation). The *historical hospitals* have often transferred the cultural heritage to third not-health entities to ensure its valorisation and usability to the public. The cultural heritage is still preserved and can be enjoyed but it lost all relations with the health purposes, being removed and dislocated from the original health context.
- *Conservation* (high administration/scarce valorisation). This quadrant of the matrix is populated by those governance models that perform the task of preserving cultural heritage but have not yet developed cultural heritage valorisation activities that go beyond mere exhibition. This is the most widespread governance model among the case studies, which has as primary objective the protection of the cultural heritage while maintaining it within health care organizations.
- *Promotion* (high administration/high valorisation). In this case, the valorisation of cultural heritage become prerequisite for defining the governance model in *historical hospitals* such as the recovery and the promotion of health. In this way, *historical hospitals* formalise their dual nature, modelling corporate strategy on these goals with a direct commitment not only to the dissemination of knowledge and enjoyment of cultural heritage but also to the integration of cultural paths in the health care activities. Moreover, in more advanced cases, this valorisation can take place by networking the *historical hospital* with other public or private (mainly health-related) cultural assets in the reference area.

The four cultural heritage governance models identified in the interviews can be placed in this matrix to show how each responds differently to the problems identified and referred to by developing a specific management strategy.

**Fig. 3:**
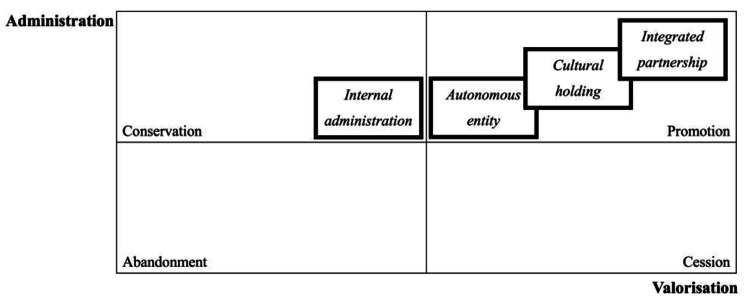
Positioning of governance models in the matrix that show the capability of response to the problems of administration and valorisation (our elaboration)

The *internal-administration* model can be easily recognised as conservation strategy. On the other hand, the *autonomous entity* model starts, as noted above, the integration of cultural strategy into health care during its valorisation activities. In addition, the network models such as *cultural holding* and *integrated partnership* favour also the integration of the managed cultural heritage into both tourism and museum routes and, at the same time, into health routes in their valorisation strategies (Fig. 3).

The matrix well explains the reasons for the choice of these governance models of the cultural heritage by the *historical hospitals* and the reasons for their attempt to move to network models. In fact, all four governance models allowed *historical hospitals* to move out of both the area of abandonment and cession of the owned cultural heritage, avoiding the problem of its dispersion.

## Conclusions

In this research, four governance models of cultural heritage owned by *historical hospitals* were identified. Their positioning within the matrix (commonly used to analyse cultural entities governance) which relates the legal nature of the decision-making organizations and the organizational levels of reference, shows that they constitute typical governance models of cultural heritage even if they are applied by health institutions.

Positioning these models in a new matrix which relates the capability of these governance solution to afford the main managing issues we can comprehend the reasons of their adoptions. These governance models allowed *historical hospitals* to move out of the areas of abandonment and of cession of the owned cultural heritage, safeguarding its presence within them (*internal administration*).

Moreover, in some cases, these models are strongly flanking preservation of these assets with their valorisation and enforcing their integration with healthcare scopes (autonomous entities). Humanization of care and art therapy could represent effective examples of integration between cultural and health purposes.

Otherwise, the networking with other public (cultural holding) or private (integrated partnership) healthcare and touristic institutions can increase the valorisation.

A limitation of the present study is the limited number of *historical hospitals* investigated, which reduces the generalization of the obtained evidence. Otherwise, the case studies selected, for the history and the complexity of their artistic assets, are probably the most representative *historical hospitals* in the world.

A possible development of this research can be the study of a wider sample of *historical hospitals* in Europe or worldwide to better verify the attitude of the identified governance models to integrate the needs of administration and valorisation of the cultural heritage owned by healthcare institutions.

### Electronic supplementary material

Below is the link to the electronic supplementary material.

Supplementary Material 1

## Data Availability

Research uses official publicly available data (www.fondazionesantamarianuova.it; www.scuolagrandesanmarco.it; https://www.policlinico.mi.it/; https://www.aslroma1.it/; www.museoartisanitarie.it).

## Declaration

### Other

The interview used in our study was developed for this study. We upload an English language version as a supplementary file.
