# Supplementary material for: Governance models for historical hospitals: evidence from Italy
Source: BMC Health Serv Res. 2024 Mar 6;24:293. doi: 10.1186/s12913-024-10640-w (PMC10918959; doi:10.1186/s12913-024-10640-w)
Supplement: Supplementary file 1 — Supplementary Material 1 [file 12913_2024_10640_MOESM1_ESM.docx]

**Appendix 1 – Scheme of semi-structured interview**

SECTION A – HOSPITAL AND REFERRAL ENTITY

- What is the configuration of the *historical hospital’s* referring entity?
- What is the current configuration of the *historical hospital*?
- Could you provide data relating to *historical hospital* activities (no. of beds, no. of employees, no. of simple and complex operating units, no. of specialty/ambulatory visits per year, no. of interventions per year, etc.)?

SECTION B – HISTORICAL NOTES

- Could you briefly trace the history of the *historical hospital* from its inception to the present, highlighting the most relevant facts?

SECTION C - CULTURAL HERITAGE

- What are the main works of art owned?
- What is the main artistic/historical/architectural heritage owned by *historical hospital*?
- Any plan for the enhancement/conversion/promotion of *historical hospital’s* cultural heritage?

SECTION D – CULTURAL HERITAGE – HEALTH INTEGRATION

- What relationship exists/should exist between *historical hospital* and health activities?
- What are the most significant experiences of cultural heritage-health integration within *historical hospital*?
- What healthcare staff/patient involvement initiatives of cultural heritage-health integration have been/are/will be put in place into *historical hospital*?
- What citizen involvement initiatives of cultural heritage-health integration have been/are/will be put in place into *historical hospital*?
- What relationships have been/are/will be forged with local institutions/schools/associations for the cultural heritage-health integration?

SECTION E – GOVERNANCE MODEL

- Could you provide the following information?
- H*istorical hospital* (legal) configuration
- Acts, Deliberations, Statute
- Recognized institutional purposes of *historical hospital*
- Relationship with local health authorities or other stakeholders

SECTION F – ORGANIZATIONAL MODEL

- Could you provide the following information?
- N° employees/collaborators/staff
- Organization chart
- Functional chart
- Usability (opening hours, organized visits, digital paths, etc.)
- Annual number of *historical hospital*’s users
- Channels used to spread knowledge about the *historical hospital*
- Communication (strategy adopted, tools employed, etc.)
- Statistics on visitors VS users

SECTION G – FINANCING

- Could you provide the following information?
- Revenue
- Expenditures
- Economic contributions by local health authorities or other stakeholders
- Planning and reporting tools
- Donations received for the historical hospital
- Donations received for health care
- Crowdfunding, fundraising, …
